# Longitudinal Population-Level Shifts in Infectious Disease–Specific Health Literacy During and After the COVID-19 Pandemic in Zhejiang, China: Six-Round Cross-Sectional Study

**DOI:** 10.2196/89958

**Published:** 2026-08-05

**Authors:** Zhao Yusui, Dingming Yao, Yue Xu, Xiujing Hu, Qingqing Wu, Lei Wang, Xuehai Zhang, Zhen Jiang

**Affiliations:** 1 Zhejiang Provincial Center for Disease Control and Prevention, 3399 Binsheng Road, Hangzhou, Zhejiang, 310051, China, 86 87115486

**Keywords:** IDSHL, COVID-19, longitudinal study, public health, China, infectious disease-specific health literacy

## Abstract

**Background:**

Infectious disease–specific health literacy (IDSHL)—the capacity to obtain, process, and understand basic infectious disease information needed to make appropriate health decisions—is a critical determinant of public health outcomes. The COVID-19 pandemic created an unprecedented global public health education context; yet, its long-term association with population-level IDSHL beyond the pandemic remains unclear. In China, stringent public health measures and digital information campaigns were implemented, yet their lasting impact on IDSHL across different disease domains has not been systematically evaluated over a full pandemic cycle.

**Objective:**

This study aimed to evaluate the longitudinal trends in IDSHL among residents of Zhejiang Province, China, from 2019 (prepandemic) to 2024 (postpandemic), and to assess whether the pandemic period was temporally associated with sustained improvements in IDSHL.

**Methods:**

Six annual cross-sectional surveys were conducted from 2019 to 2024 across 30 counties in Zhejiang Province using consistent multistage stratified random sampling. A validated 12-item IDSHL questionnaire assessed knowledge, behavior, and skills. Annual sample sizes ranged from 17,131 to 19,257 (N=112,917). Data were weighted to the 2020 census population by age and urban or rural residence. Joinpoint regression identified inflection points in the annual proportion of residents with adequate IDSHL (score≥12). Multivariate logistic regression estimated adjusted odds ratios (ORs) for adequate IDSHL by year, with 2021 as the reference (the peak acute pandemic phase), controlling for sociodemographics. All analyses incorporated sampling weights.

**Results:**

Population-weighted overall IDSHL scores increased from 2019 (mean 10.46 SD 3.09) to 2024 (mean 11.40 SD 2.88*; P*<.001). All subscale scores (knowledge, behavior, and skills) showed upward trends (all *P*<.001). Joinpoint regression revealed a rapid annual increase in adequate IDSHL during 2019-2021 (annual percent change 9.42%, 95% CI 6.10‐13.45; *P*<.001), which slowed post-2021 (annual percent change 2.28%, 95% CI –0.38 to 3.73; *P*=.07). Correct response rates surged for pandemic-salient items (eg, respiratory etiquette from 28.44% to 39.67%), while hepatitis B transmission knowledge fluctuated (63.28% in 2019, 60.71% in 2022, 63.84% in 2024), indicating that not all non–COVID-19 content improved monotonically. Adjusted logistic regression showed the lowest odds of adequate IDSHL in 2019 (OR 0.74, 95% CI 0.71‐0.78) and highest in 2024 (OR 1.10, 95% CI 1.05‐1.15) compared to 2021.

**Conclusions:**

The COVID-19 pandemic was temporally associated with significant and sustained population-level improvements in IDSHL, particularly for pandemic-relevant knowledge and behaviors. However, gains were time-sensitive and not uniform across all disease domains or demographic groups. These findings underscore the need for postpandemic health strategies that reinforce comprehensive IDSHL through sustained education and address digital divides to bridge persistent equity gaps. Policymakers should prioritize low digital-literacy populations through hybrid (offline+online) educational interventions, and sustain routine surveillance of IDSHL to detect declines in nonpandemic disease knowledge. This study is the first to document six-year population-level IDSHL trends across the full pandemic cycle, providing evidence for future crisis communication and routine health promotion.

## Introduction

Infectious disease–specific health literacy (IDSHL)—defined as the cognitive, behavioral, and skills-based capacity to obtain, process, and understand basic infectious disease information needed to make appropriate health decisions—is a critical social determinant of health outcomes [1,2]. It empowers individuals to engage in preventive behaviors, adhere to treatment regimens, and navigate health systems effectively [3,4]. The COVID-19 pandemic, caused by SARS-CoV-2, placed unprecedented emphasis on public understanding of transmission dynamics, nonpharmaceutical interventions (NPIs), and vaccination [5-7]. For nearly three years, governments worldwide, including China, implemented rigorous containment strategies and launched intensive public health communication campaigns [8,9]. The pandemic evolution in China was broadly characterized by experts into several distinct phases: the prepandemic era (2019), the acute pandemic phase (2020‐2022, characterized by stringent NPIs and intensive public health messaging), and the initial postpandemic period (2023‐2024, following the transition to endemic management in January 2023) [10-13]. This phased approach provides a structured framework to examine the differential impacts of varying intensities of public health interventions on IDSHL over time.

This period offered a unique opportunity to examine population-level changes in IDSHL in response to a major, sustained public health crisis. By capitalizing on this unique context, we aimed to assess whether a shift in IDSHL was observed during and after the pandemic. It is particularly noteworthy that during the COVID-19 pandemic, digital platforms, and social media (eg, WeChat, Weibo, and news applications) became the central channels through which the public accessed authoritative pandemic updates, health guidance, and subsequently formed health-related behaviors [14,15]. This shift created a highly digitalized health information environment. Consequently, the observed changes in public IDSHL captured by our consecutive surveys coincide with the period of intense digital information exposure, during which public cognition and behavior were shaped by large-scale digital health messaging.

It is crucial to emphasize that this study uses an observational, repeated cross-sectional design without a control group. Therefore, while we document strong temporal associations between the pandemic phases and changes in IDSHL, we cannot infer causality. All interpretations of “association,” “temporal relationship,” or “observed change” refer to statistical associations at the population level.

Prior to the pandemic, studies indicated suboptimal levels of IDSHL in various populations, with significant gaps in knowledge about both common and emerging infections [16]. Research from our previous work using 2019 and 2022 data suggested potential improvements in IDSHL, especially for respiratory pathogens, but was limited to two time points [17]. Understanding whether the seismic event of COVID-19 was temporally associated with a transient or lasting shift in the public’s IDSHL is crucial [18]. A sustained elevation would suggest that major public health crises may coincide with durable positive changes in population health competence. Conversely, a reversion to baseline or uneven progress would highlight the need for more targeted, persistent educational efforts beyond crisis periods.

Based on a unique longitudinal dataset comprising six consecutive annual surveys (2019‐2024) from Zhejiang Province, China, we propose a temporal reconceptualization for analysis. The timeline is divided into three distinct phases aligned with fundamental shifts in the pandemic response focus: stage 1: the prepandemic era (2019), stage 2: the acute pandemic phase (2020‐2022), and stage 3: the initial postpandemic period (2023‐2024). This phased framework corresponds directly with the annual measurement points, thereby enabling a clearer examination of the longitudinal patterns associated with a major public health crisis.

Our primary objectives were to (1) describe longitudinal trends in overall and domain-specific IDSHL scores, (2) examine changes in correct response rates for specific IDSHL items, and (3) assess temporal changes in the proportion and adjusted odds of having adequate IDSHL across the prepandemic, acute pandemic, and postpandemic phases. We hypothesized that the pandemic would be temporally associated with a stepwise and sustained increase in IDSHL, with larger gains for pandemic-salient content (eg, respiratory etiquette and vaccination) and persistent disparities across sociodemographic groups.

## Methods

### Study Design

This study used a repeated cross-sectional design, conducting independent surveys annually from 2019 to 2024. Given the observational, repeated cross-sectional design without a control group, we interpret our findings as population-level associations over time, not causal effects.

### Setting

The surveys were conducted in Zhejiang Province, a major coastal province in eastern China with a diverse population of over 64 million. The formal survey was conducted annually between August 1 and October 31 of each year from 2019 to 2024. Recruitment and data collection occurred simultaneously during this period. The surveys were carried out within 30 representative counties or districts surveillance sites across the province [19]. These sites were selected using stratified cluster random sampling based on per capita gross domestic product (tertiles) to ensure representativeness. The methods for sampling, sample size determination, and data collection were consistent with those described in our previously published two-round cross-sectional study using the 2019 and 2022 data from the same surveillance system [17]. This study extends this work by including four additional survey rounds (2020, 2021, 2023, and 2024) to cover the full pandemic cycle from prepandemic to postpandemic periods.

### Participants

#### Inclusion and Exclusion Criteria

Eligibility criteria were (1) age 15‐69 years, (2) ability to communicate in Mandarin or local dialect, and (3) permanent resident status (accessibility). No additional exclusion criteria were applied. There were no restrictions based on sex, ethnicity, education, or other demographic characteristics.

#### Sampling Procedures

A multistage stratified random sampling method was consistently applied. The sampling procedure followed five stages, as detailed in our previous publication [17]: (1) county selection: 30 counties and districts were selected from the 90 counties in Zhejiang Province using stratified cluster random sampling based on per capita gross domestic product (tertiles); (2) township selection: within each selected county, 4 townships were randomly selected; (3) segment (residential block) selection: within each township, 2 residential segments were randomly selected; (4) household selection: approximately 100 households were randomly selected from a complete address list within each segment; and (5) individual selection: one permanent resident aged 15‐69 years per household was selected using a Kish grid.

#### Participant Characteristics

Participants were permanent residents of Zhejiang Province aged 15‐69 years. Across the six annual surveys (N=112,917), approximately 52% (n=58,717) were female and 48% (n=54,200) were male each year. The sample included participants with education levels ranging from primary school or lower (about 27%, 30,487/112,917 to 36%, 40,650/112,917 across years) to undergraduate or higher (less than 1% in each year). Farmers were the largest occupational group (35,004/112,917, 31%-51,941/112,917, 46% across years), with a decreasing trend over time.

#### Study Size, Power, and Precision

The target sample size was determined using the same method as our previous study [17]. Briefly, the sample size per county was calculated using the formula:


$$N\text{=}\frac{{µ}_{\alpha }^{2}\times p(1\text{-}p)}{\delta^{2}}\times deff$$

We set α=.05 (hence μ_α_=1.96), used the 2018 health literacy rate for Zhejiang (26.24%) as the expected prevalence p, allowed a maximum error *δ*=0.03936, and took a design effect deff=1 for simple random sampling. After accounting for an anticipated 25% rate of invalid questionnaires or refusals, the final target sample size per county was 640. With 30 counties, the total annual target sample size was approximately 19,200. No formal a priori power analysis for detecting specific effect sizes was conducted, as the primary goal was precise annual surveillance rather than hypothesis testing of a single a priori effect. The achieved annual sample sizes ranged from 17,131 to 19,257 across years. Precision was quantified using 95% CI for all point estimates (eg, proportions, odds ratios [ORs], annual percent changes [APC]).

## Measures and Covariates

### Outcome, Exposures, and Covariates

The primary outcome was adequate IDSHL, defined as a score of ≥12 on the IDSHL scale (see Instrumentation; Multimedia Appendix 1). The main exposure of interest was survey year (2019‐2024), which served as a proxy for the pandemic phase (prepandemic, acute pandemic, and postpandemic). Covariates included sex, age group (15-29, 30-39, 40-49, 50-59, and 60-69), ethnicity (Han vs minority), education level (primary school or lower, middle school, high school, technical school or college, undergraduate or higher), marital status (married vs unmarried, divorced, or widowed), occupation (farmer, worker, agency or institutional personnel, student, and other), and urban or rural residence. All covariates were self-reported and treated as potential confounders in multivariable analyses.

### Data Collection

Data were collected via household visits using tablet computers. Prior to household visits, community workers or community health physicians (investigators) contacted the sampled household head by telephone, informed them of the selected family member, and scheduled a face-to-face visit. If the appointment failed, another was scheduled. If three consecutive attempts to schedule a successful visit failed, the household was replaced with the next household on the sampling list, and the recruitment process was restarted. Prior to the formal survey, both the surveyor and the quality control supervisor verified whether the participating family member matched the randomly selected subject. If not, the correct member was requested. If the selected member was unavailable, a new appointment was scheduled via telephone.

Questionnaire administration followed 2 modes: if the respondent was capable of self-completion, the surveyor remained present to provide assistance and correct obvious errors in basic information. If the respondent could not complete the questionnaire independently, the surveyor administered it through face-to-face interviewing.

### Quality of Measurements

To ensure data authenticity and quality, the following measures were implemented.

#### Training

All surveyors, supervisors, and quality control personnel underwent a standardized training program consisting of collective lectures, on-site demonstrations, role-playing or simulation exercises, and assessments based on presurvey pilot tests. Unified technical manuals and standardized teaching materials were used. Trainers were staff members from the Zhejiang Provincial Center for Disease Control and Prevention, each with over 5 years of experience in IDSHL monitoring.

#### Audio Recording

Audio recording was activated throughout each survey session.

#### Photography

After the survey, the surveyor took photographs of the setting for subsequent verification.

#### Multidimensional Quality Checks

Supervisors reviewed audio recordings, survey duration, and on-site photos. A questionnaire was deemed invalid and replaced with a new household if (1) leading questions were present in the audio recording, (2) the survey duration was less than 10 minutes (a threshold established during pilot testing as the minimum reasonable time), and (3) there was an inconsistency between on-site photos and actual survey conditions.

#### Electronic Information System

The entire process—questionnaire administration, data collection, storage, and quality control—was managed through an integrated electronic information system, ensuring data consistency and integrity.

### Instrumentation

IDSHL was assessed using a validated 12-item subscale from the National Health Literacy Surveillance Survey in China [17]. The scale covers 3 dimensions: knowledge (6 items), behavior (4 items), and skills (2 items). It includes one true or false item, 8 single-choice items, and 3 multiple-choice items. Correct answers to multiple-choice questions were awarded 2 points, while single-choice and true or false items received 1 point each, for a total score of 15. Participants scoring ≥12 points (≥80%) were classified as having “adequate IDSHL” [19].

This cutoff has been widely used in China’s National Health Literacy Surveillance System and is recommended in the official surveillance guidelines [17].

### Masking

Given the observational, cross-sectional survey design, no formal blinding or masking procedures were implemented. Surveyors were trained to administer the questionnaire in a standardized manner without revealing the study hypotheses or influencing responses. Participants were not informed of specific research hypotheses beyond the general purpose of the health literacy survey. No experimental manipulation or group assignment occurred, so masking of condition assignments was not applicable.

### Psychometrics

All 12 items demonstrated satisfactory content validity, with indices greater than 0.8. The overall internal consistency of the scale was acceptable (Cronbach α=0.67). For the 3 subscales, Cronbach α values were 0.71 for knowledge, 0.65 for behavior, and 0.63 for skills. Given the scale’s multidimensionality and diverse item content (covering multiple diseases and response formats), these coefficients are considered adequate for population-level surveillance [17]. Reliability estimates are based on the current sample (N=112,917). No additional validity coefficients from external samples are reported, as the scale is a national surveillance instrument with established content validity.

### Conditions and Design

This was a nonexperimental (observational) design with no manipulated conditions. The study used a repeated cross-sectional design (six annual waves, 2019‐2024). No randomization or intervention assignment occurred. The primary comparison was across time (survey year) as a proxy for pandemic phases.

### Data Diagnostics

Planned data diagnostics included the following prespecified criteria: (1) post data collection exclusion of participants was based solely on the 3 quality control criteria (leading questions, duration <10 min, and photo inconsistency); no additional exclusion criteria were applied. (2) Missing data: less than 2% of sampled households were replaced due to quality issues; no imputation was performed for missing item responses as the questionnaire was reviewed for completeness on-site. We conducted Little Missing Completely At Random (MCAR) test on the full dataset (N=112,917) using full information maximum likelihood. The result was nonsignificant (*χ*²_145_=156.73; *P*=.24), indicating that missing data (at the household replacement level) were missing completely at random (degrees of freedom derived from the number of variables and observed missing patterns). (3) Outliers: no statistical outlier removal was performed; all valid responses were retained. (4) Data distributions: normality of IDSHL scores was assessed visually using histograms and Q-Q plots; for nonnormal distributions, nonparametric tests (Kruskal-Wallis) were used as specified. (5) Data transformations: no data transformations were applied.

### Analytic Strategy

Data were analyzed using SPSS (version 27.0; IBM Corp) and Joinpoint Regression Program (version 5.4.0; National Cancer Institute). Descriptive statistics were presented as frequencies (percentages) for categorical variables and means (SDs) for continuous variables. No qualitative analysis was performed.

#### Weighting

To ensure the representativeness of the provincial estimates, all analyses (descriptive, ANOVA, Kruskal-Wallis, Joinpoint, and logistic regression) incorporated sampling weights. Weights were calculated as the inverse probability of selection, poststratified to the 2020 Zhejiang census population distribution by age group (15-29, 30-39, 40-49, 50-59, and 60-69 years) and urban or rural residence [20].

#### Group Comparisons

The Kruskal-Wallis H test was used to compare distributions of sociodemographic characteristics and correct response rates for individual items across the 6 years due to nonnormality. One-way ANOVA was used to compare weighted mean IDSHL scores (overall and subscales) across years, with post hoc least significant difference (LSD) tests for pairwise comparisons. No adjustment for multiple comparisons was applied to least significant difference tests, as these were considered exploratory; however, the overall ANOVA *F* test protected the type I error rate at the .05 level for the primary hypothesis of any yearly difference.

#### Joinpoint Regression

Joinpoint regression was used to model the temporal trend in the weighted annual proportion of residents with adequate IDSHL from 2019 to 2024 and to identify significant inflection points. The maximum number of joinpoints was set to 1 given the limited number of time points. The APC and average annual percent change (AAPC) with 95% CIs were calculated.

#### Logistic Regression

Multivariate logistic regression was performed with “adequate IDSHL” (score≥12) as the dependent variable. The year 2021 was chosen as the reference category because it represents the peak of the acute pandemic phase in Zhejiang Province (characterized by the “Dynamic COVID-zero” strategy and intensive public health messaging). Joinpoint regression identified 2021 as a significant inflection point. All models adjusted for sex, age group, ethnicity, education, marital status, and occupation. Sampling weights were applied using SPSS Complex Samples module. A two-tailed *P* value of <.05 was considered statistically significant.

#### Relationship Between Joinpoint and Logistic Regression Results

The Joinpoint results describe the trajectory of the population prevalence of adequate IDSHL, while the logistic regression quantifies the individual-level adjusted odds for each year relative to 2021. Together, they show a consistent pattern: a sharp initial increase from 2019 to 2021 (lowest OR in 2019, steep positive APC), followed by a plateau from 2021 onwards (nonsignificant ORs for 2022‐2024 compared to 2021, and a nonsignificant APC after 2021). Missing data were minimal (<2% of sampled households replaced due to quality issues). No imputation was performed.

### Ethical Considerations

#### Ethics approval

The study protocol was approved by the Ethics Committee of the Zhejiang Provincial Center for Disease Control and Prevention (approval No. 2022-027-01 for the 2019 and 2022 surveys; subsequent annual surveys were conducted under the same ethical approval with protocol amendments approved by the committee). The study was conducted in accordance with the principles of the Declaration of Helsinki.

#### Informed consent

Written informed consent was obtained from all participants prior to the survey. Participants were informed about the purpose of the study, the procedures (including audio recording and photography for quality control), the voluntary nature of participation, and their right to withdraw at any time. The consent form explicitly covered the use of anonymized data for research and publication.

#### Privacy and confidentiality

All data were anonymized prior to analysis. No personal identifiers (eg, names, ID numbers, and exact addresses) were retained in the analytic dataset. Audio recordings and photographs were used solely for real-time quality control and were deleted upon completion of data validation. Access to the deidentified data was restricted to the research team.

#### Compensation

Upon completion of the survey, each participant received a small gift valued at approximately 50 RMB (about US $7) as compensation for their time.

#### Identifiable images

**Figure 1.**
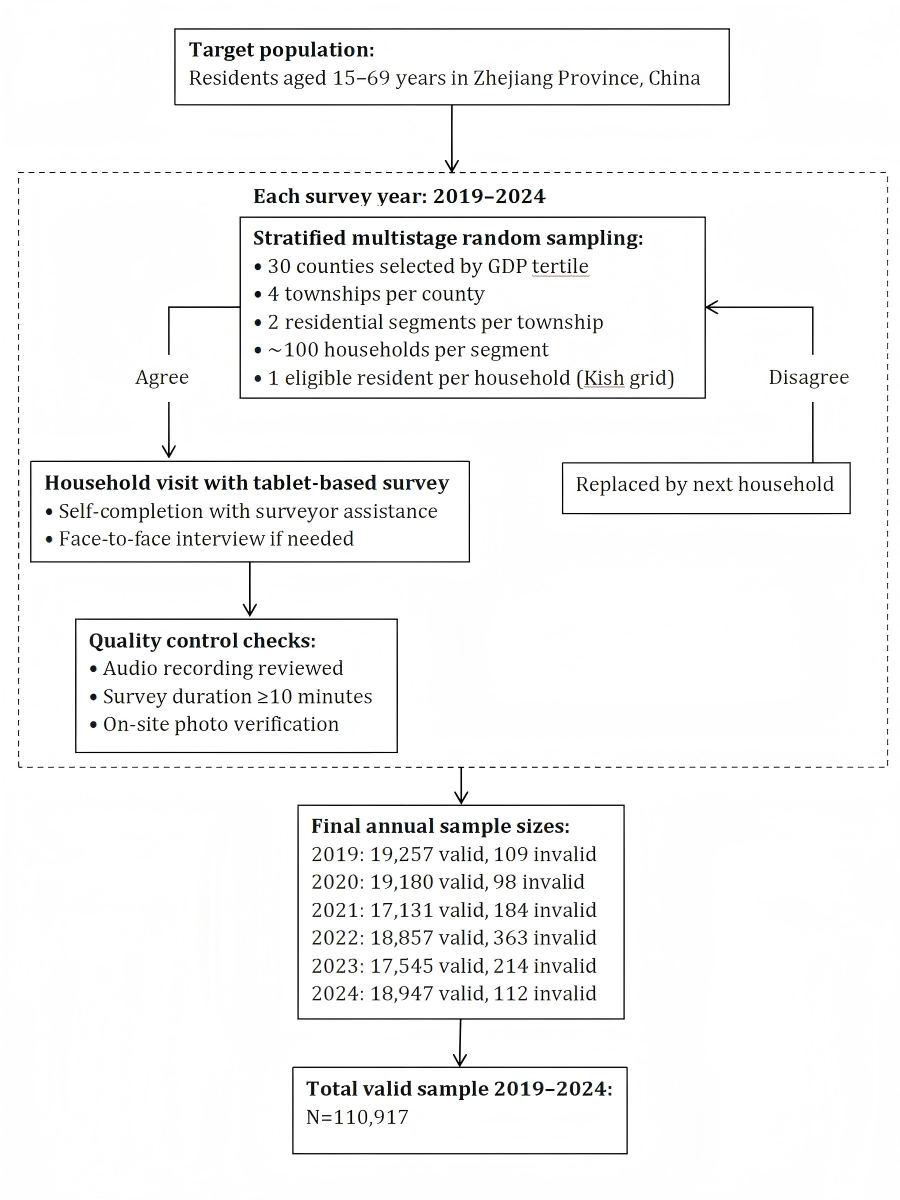
Participant flow diagram, showing the number of households contacted, eligible, and finally included for each survey year. GDP: gross domestic product.

Figure 1 (participant flow diagram) contains no identifiable individual images. No photographs or images of individual participants are included in the paper or supplementary materials.

## Results

### Participant Flow

A total of 112,917 valid questionnaires were analyzed across 6 years. Figure 1 presents the participant flow diagram, showing the number of households contacted, eligible, and finally included for each survey year. Across the 6 annual surveys (2019‐2024), the number of valid participants ranged from 17,131 (2021) to 19,257 (2019). Replacement of invalid questionnaires due to quality issues (leading questions, duration<10 min, and photo inconsistency) occurred in less than 2% of sampled households per year; no further exclusions were applied.

### Recruitment

Recruitment and data collection occurred annually between August 1 and October 31 of each year from 2019 to 2024, as described in the Setting subsection.

### Participant Characteristics

Table 1 presents the detailed sociodemographic characteristics of participants across the six annual surveys (2019‐2024). Significant differences were observed across years in sex, age, education, and occupation distributions (all *P*<.001), highlighting the need for weighting. Weighting was applied to all subsequent analyses.

**Table 1.** Sociodemographic characteristics of participants in six annual cross-sectional surveys (2019‐2024), Zhejiang Province, China (N=110,917).

Content and group		2019 Group (n=19,257), n (%)	2020 Group (n=19,180), n (%)	2021 Group (n=17,131), n (%)	2022 Group (n=18,857), n (%)	2023 Group (n=17,545), n (%)	2024 Group (n=18,947), n (%)	H^a^ (*df*)	*P* value
Sex								23.45 (6)	<.001
Male		9175 (47.65)	9298 (48.48)	8020 (46.82)	8762 (46.47)	8237 (46.95)	8807 (46.48)		
Female		10,082 (52.35)	9882 (51.52)	9111 (53.18)	10,095 (53.53)	9308 (53.05)	10,140 (53.52)		
Ethnicity								9.32 (6)	.10
Han		19,050 (98.93)	18,917 (98.63)	16,909 (98.70)	18,634 (98.82)	17,342 (98.84)	18,702 (98.71)		
Minority		207 (1.07)	263 (1.37)	222 (1.30)	223 (1.18)	203 (1.16)	245 (1.29)		
Marital status								256.14 (6)	<.001
Unmarried, divorced, or widowed		3214 (16.69)	3062 (15.96)	2652 (15.48)	3162 (16.77)	3374 (19.23)	3898 (20.57)		
Married		16,043 (83.31)	16,118 (84.04)	14,479 (84.52)	15,695 (83.23)	14,171 (80.77)	15,049 (79.43)		
Age group (years)								78.05 (20)	<.001
15‐29		1370 (7.11)	1534 (8.00)	1278 (7.46)	1529 (8.11)	1568 (8.94)	1624 (8.57)		
30‐39		2313 (12.01)	2486 (12.96)	2470 (14.42)	2626 (13.93)	2354 (13.42)	2540 (13.41)		
40‐49		2749 (14.28)	3890 (20.28)	3502 (20.44)	3218 (17.07)	3361 (19.16)	3301 (17.42)		
50‐59		6517 (33.84)	6001 (31.29)	5508 (32.15)	6268(33.24)	5245 (29.89)	5546 (29.27)		
60‐69		6308(32.76)	5269 (27.47)	4373 (25.53)	5216(27.66)	5017 (28.60)	5936 (31.33)		
Education								751.61 (20)	<.001
Primary school or lower		7020 (36.45)	6643 (34.64)	5394 (31.49)	5887 (31.22)	4863 (27.72)	5197 (27.43)		
Middle school		6196 (32.18)	6580 (34.31)	5735 (33.48)	6254 (33.17)	5974 (34.05)	6313 (33.32)		
High school		3130 (16.25)	3000 (15.64)	2906 (16.96)	3134 (16.62)	3057 (17.42)	3399 (17.94)		
Technical school or college		2848 (14.79)	2888 (15.06)	3015 (17.60)	3480 (18.45)	3539 (20.17)	3937 (20.78)		
Undergraduate or higher		63 (0.33)	69 (0.36)	81 (0.47)	102 (0.54)	112 (0.64)	101 (0.53)		
Occupation								931.19 (20)	<.001
Farmers		8738 (45.38)	8979 (46.81)	6667 (38.92)	7600 (40.30)	6466 (36.85)	5945 (31.38)		
Workers		2581 (13.40)	2451 (12.78)	2409 (14.06)	2681 (14.22)	2840 (16.19)	3326 (17.55)		
Agency or Institutional personnel^b^		4565 (23.71)	4786 (24.95)	4855 (28.34)	5175 (27.44)	5179 (29.52)	5873 (31.00)		
Students		671 (3.48)	530 (2.76)	251 (1.47)	442 (2.34)	586 (3.34)	514 (2.71)		
Other^c^		2702 (14.03)	2434 (12.69)	2949 (17.21)	2959 (15.69)	2474 (14.10)	3289 (17.36)		
Total		19,257(100)	19,180(100)	17,131(100)	18,857(100)	17,545(100)	18,947(100)	—^d^	—

a The test statistic H refers to the Kruskal-Wallis H statistic.

b “Agency or Institutional personnel” refers to people working in state organs, state-owned enterprises, institutions, and other public roles.

c The “Other” category includes unemployed people and those with occupations other than those already listed.

d Not applicable.

### Statistics and Data Analysis

#### Item-Specific Correct Response Rates

Table 2 presents correct response rates for each IDSHL item. Most items showed significant increases over time (*P*<.001). The most dramatic rise was for respiratory etiquette (cough or sneeze), from 28.44% (n=5476) in 2019 to 39.67% (n=7516) in 2024, with a sharp jump in 2020 (n=7438, 38.78%). Hepatitis B transmission knowledge followed a U-shaped pattern: 63.28% (n=12,186; 2019), 60.71% (n=11,448; 2022), and 63.84% (n=12,096; 2024). Other items, such as fever management and vaccination contraindications, also improved steadily. For example, correct responses for vaccination contraindications increased from 80.69% (n=15,538) in 2019 to 85.35% (n=16,171) in 2024.

**Table 2.** Correct response rates for individual IDSHL^a^ items across six survey years, 2019‐2024.

Types and question	2019 Year N(%)	2020 Year N(%)	2021 Year N(%)	2022 Year N(%)	2023 Year N(%)	2024 Year N(%)	*t* test (*df*)	*P* value
True or false question								<.001
The best way to prevent flu is to take antibiotics (anti-inflammatories).	10,760 (55.88)	11,570 (60.32)	10,226 (59.69)	10,912 (57.87)	10,100 (57.57)	10,738 (56.67)	113.45 (5)	
Single-choice questions								
In which of the following ways can hepatitis B be transmitted to others?	12,186 (63.28)	11,923 (62.16)	10,684 (62.37)	11,448 (60.71)	11,128 (63.43)	12,096 (63.84)	52.11 (5)	
For the treatment of tuberculosis patients, which of the following statements is correct?	12,051 (62.58)	12,470 (65.02)	10,918 (63.73)	12,164 (64.51)	11,762 (67.04)	12,590 (66.45)	112.26 (5)	
In which of the following situations should vaccination of children be suspended?	15,538 (80.69)	15,803 (82.39)	14,399 (84.05)	15,960 (84.64)	14,946 (85.19)	16,171 (85.35)	231.72 (5)	
If you have a fever, which of the following is correct?	15,097 (78.40)	15,737 (82.05)	14,333 (83.67)	15,755 (83.55)	14,209 (80.99)	15,172 (80.08)	259.78 (5)	
If a virulent infectious disease occurs in a certain place, which of the following practices is correct?	15,812 (82.11)	16,510 (86.08)	14,936 (87.19)	16,464 (87.31)	15,286 (87.12)	16,458 (86.86)	320.03 (5)	
Open windows frequently for ventilation during flu season. Regarding window ventilation, which of the following statements is correct?	14,188 (73.68)	14,138 (73.71)	12,673 (73.98)	14,017 (74.33)	13,182 (75.13)	14,603 (77.07)	84.58 (5)	
What is the correct way to read body temperature with a glass thermometer?	10,060 (52.24)	10,564 (55.08)	9505 (55.48)	10,618 (56.31)	10,383 (59.18)	11,564 (61.03)	375.09 (5)	
If you are bitten by a dog but not seriously, what is the right thing to do?	18,669 (96.95)	18,555 (96.74)	16,646 (97.17)	18,315 (97.13)	17,108 (97.51)	18,506 (97.67)	41.31 (5)	
Multiple-choice questions								
What should parents do when their children have symptoms such as fever and a rash?	13,171 (68.40)	13,389 (69.81)	12,179 (71.09)	13,629 (72.28)	12,919 (73.63)	14,418 (76.10)	356.29 (5)	
When sick and dead livestock are found, which of the following practices is correct?	14,897 (77.36)	14,961 (78.00)	13,417 (78.32)	15,128 (80.22)	14,244 (81.19)	15,863 (83.72)	336.76 (5)	
When coughing or sneezing, which of the following is correct?	5476 (28.44)	7438 (38.78)	6830 (39.87)	7306 (38.74)	6820 (38.87)	7516 (39.67)	793.88 (5)	

a IDSHL: infectious disease-specific health literacy.

#### Overall and Domain-Specific IDSHL Scores

Weighted mean total IDSHL score increased from 10.46 (SD 3.09) in 2019 to 11.40 (SD 2.88) in 2024 (*P*<.001). All subscales (knowledge, behavior, and skills) showed significant upward trends (all *P*<.001), with behavior exhibiting the largest increase, particularly in 2020 (Table 3).

**Table 3.** Weighted mean (SD) scores for IDSHL^a^ overall and subscales, 2019‐2024.

Groups	Knowledge, mean (SD)	Behavior, mean (SD)	Skills, mean (SD)	Overall, mean (SD)
2019	4.41 (1.54)	4.62 (1.67)	1.44 (0.65)	10.46 (3.09)
2020	4.49 (1.52)	4.92 (1.69)	1.49 (0.62)	10.90 (3.09)
2021	4.53 (1.49)	5.02 (1.67)	1.51 (0.61)	11.07 (3.07)
2022	4.56 (1.47)	5.04 (1.63)	1.52 (0.61)	11.12 (2.99)
2023	4.64 (1.45)	5.05 (1.62)	1.55 (0.60)	11.24 (2.95)
2024	4.68 (1.40)	5.15 (1.60)	1.57 (0.59)	11.40 (2.88)
*F* test (*df*)	47.566 (5)	157.740 (5)	99.397 (5)	141.230 (5)
*P* value	<.001	<.001	<.001	<.001

a IDSHL: infectious disease-specific health literacy.

#### Joinpoint Regression of Adequate IDSHL Proportion

Joinpoint regression describes population-level annual prevalence trends, whereas logistic regression estimates individual-level adjusted odds for each year relative to 2021. Joinpoint regression identified a significant inflection point in 2021 (Table 4). From 2019 to 2021, the proportion of residents with adequate IDSHL increased sharply at an APC of 9.42% (95% CI 6.10‐13.45; *P*<.001). From 2021 to 2024, the increase slowed to an APC of 2.28% (95% CI –0.38 to 3.73; *P*=.07). Over the entire period, AAPC was 5.08% (95% CI 3.98‐6.44; *P*<.001).

**Table 4.** Joinpoint regression analysis of trends in weighted proportion of residents with adequate IDSHL^a^, Zhejiang, 2019‐2024.

Model and time period (year)		APC^b^ or AAPC^c^ (%)	95% CI	*P* value
Segment trends, APC				
2019‐2021		9.42	6.10 to 13.45	<.001
2021‐2024		2.28	-0.38 to 3.73	.07
Overall trend, AAPC				
2019‐2024		5.08	3.98 to 6.44	<.001

a IDSHL: infectious disease-specific health literacy.

b APC: annual percent change.

c AAPC: average annual percent change.

#### Multivariate Logistic Regression

Table 5 shows adjusted ORs for adequate IDSHL. Higher education was the strongest predictor, while older age (50+ years) and minority ethnicity were associated with lower odds. Compared to 2021 (reference), the odds were significantly lower in 2019 (OR 0.74, 95% CI 0.71‐0.78) and significantly higher in 2024 (OR 1.10, 95% CI 1.05‐1.15). No significant differences were found for 2020, 2022, or 2023.

**Table 5.** Multivariate logistic regression of factors associated with adequate IDSHL^a^.

Variables and categories		B	SE	Wald	*P* value	OR^b^	95% CI
Sex							
Male (reference)		0.05	0.01	15.00	<.001	1.06	1.03 to 1.08
Age group (years)^c^							
30‐39		0.02	0.04	0.20	.66	1.02	0.95 to 1.09
40‐49		0.04	0.04	1.00	.32	1.04	0.97 to 1.11
50‐59		−0.34	0.04	92.70	<.001	0.72	0.67 to 0.77
60‐69		−0.68	0.04	350.98	<.001	0.51	0.47 to 0.55
Ethnicity							
Minority (reference)		−0.20	0.06	10.41	.001	0.82	0.73 to 0.93
Education^d^							
Middle school		0.68	0.02	1371.63	<.001	1.98	1.91 to 2.05
High school		1.26	0.02	2964.03	<.001	3.52	3.36 to 3.68
Technical school or college		1.98	0.03	4797.55	<.001	7.23	6.84 to 7.65
Undergraduate or higher		2.49	0.13	394.10	<.001	12.10	9.46 to 15.47
Marital status							
Unmarried, divorced, or widowed (reference)		0.22	0.02	111.50	<.001	1.25	1.20 to 1.30
Occupation^e^							
Workers		0.05	0.02	5.40	.020	1.05	1.01 to 1.09
Agency or Institutional personnel		0.22	0.02	111.95	<.001	1.25	1.20 to 1.30
Students		0.36	0.05	48.17	<.001	1.43	1.29 to 1.58
Other		0.16	0.02	58.08	<.001	1.17	1.12 to 1.22
Year^f^							
2019		−0.30	0.02	158.01	<.001	0.74	0.71 to 0.78
2020		0.002	0.02	0.007	.94	1.00	0.96 to 1.05
2022		0.02	0.02	0.66	.42	1.02	0.97 to 1.07
2023		0.04	0.02	2.13	.14	1.04	0.99 to 1.09
2024		0.09	0.02	15.58	<.001	1.10	1.05 to 1.15
Constant		−0.98	0.08	164.74	<.001	0.38	—^g^

a IDSHL: infectious disease–specific health literacy.

b OR: odds ratio.

c Reference range for age: 15-29 years.

d Primary school or lower (reference).

e Farmers (reference).

f 2021 (reference).

g Not applicable.

## Discussion

### Support of Original Hypotheses

This 6-year study, leveraging repeated cross-sectional data from before, during, and after the COVID-19 pandemic, demonstrates that the pandemic period was temporally associated with a significant and sustained population-level elevation in IDSHL among residents of Zhejiang Province, China. We observed a sustained population-level elevation in IDSHL, with the lowest likelihood of adequate literacy occurring in the prepandemic year (2019) and the highest in the postpandemic year (2024). The most rapid gains were temporally associated with the acute pandemic phase (2019‐2021), after which the increase decelerated. The temporal pattern showed a sharper increase during the acute pandemic phase (2019‐2021) followed by a slower increase thereafter. Improvements were domain- and disease-specific: the largest gains occurred for pandemic-salient behaviors (eg, respiratory etiquette), while nonrespiratory diseases such as hepatitis B exhibited more complex, fluctuating patterns. Persistent disparities by age, education, and ethnicity indicated that the benefits associated with the pandemic were not uniformly distributed. Crucially, our observational design cannot establish causality; all interpretations refer to population-level temporal associations.

### Similarity of Results

Our findings align with previous research showing reduced incidence of other infectious diseases during the pandemic [16] and improvements in respiratory-related health literacy [14,21]. The sharp increase in adequate IDSHL during 2019‐2021 coincides with the period of most intensive public health messaging, stringent nonpharmaceutical interventions, and peak societal attention to infectious disease prevention [12]. This pattern is consistent with evidence that large-scale health communication campaigns can be temporally associated with rapid improvements in targeted knowledge and behaviors [22]. The subsequent deceleration after 2021, as the pandemic transitioned to endemic management, suggests that crisis-driven momentum is not self-sustaining—a temporal pattern not previously documented in the health literacy literature. The U-shaped pattern for hepatitis B knowledge is suggestive of a potential “crowding-out” effect described in prior literature [17,23]. However, unlike earlier work, our six-wave design revealed that not all non–COVID-19 content stagnated; items with stable, long-standing messaging (eg, vaccination contraindications) improved steadily [24].

### Interpretation

The dramatic increase in correct responses for respiratory etiquette, a direct target of COVID-19 messaging, illustrates the focused impact of crisis-driven education [25]. In contrast, the U-shaped pattern for hepatitis B knowledge implies that intense focus on a novel pathogen may temporarily divert public attention from endemic threats, though knowledge may recover as the crisis abates [26]. Notably, items unrelated to respiratory illnesses but with consistent messaging (eg, vaccination contraindications) improved steadily, indicating that generalizable knowledge can still accrue during a pandemic. These nuanced patterns highlight a key limitation of crisis-driven education: it powerfully enhances literacy in highlighted areas but does not automatically generalize across all infectious diseases.

Despite overall improvements, preexisting disparities by age, education, and ethnicity persisted. This finding echoes evidence that health literacy initiatives often benefit more advantaged groups to a greater extent [27,28]. The pandemic’s heavy reliance on digital platforms (eg, WeChat, news apps) for information dissemination may have exacerbated these gaps, as older adults and less-educated groups face documented barriers to digital access and literacy [29]. Although this study did not directly measure digital health literacy, prior literature has established its mediating role [30]. Therefore, postpandemic strategies should consider addressing digital divides through age-friendly interfaces, community-based outreach, and tailored messaging. Our data do not directly examine causal mechanisms, but personal experience of COVID-19 infection, including long COVID, could have contributed to knowledge and behavior change [31], a hypothesis warranting future investigation

### Generalizability (External Validity)

The study was conducted in a single developed Chinese province (Zhejiang) with a population of over 64 million, which limits direct generalizability to other regions or countries with different pandemic responses, health systems, or cultural contexts. However, the sampling strategy (multistage stratified random sampling, weighting to census) supports generalizability within Zhejiang Province. The findings may be most applicable to other economically developed regions in China or similar settings with intensive digital information environments. Ecological validity is supported by the real-world pandemic context and the use of routine surveillance methods.

### Limitations

Several limitations should be considered. First, the repeated cross-sectional design captures population trends but cannot follow individuals over time or establish causality. Unmeasured confounding factors (eg, other policy changes and media coverage) may have influenced IDSHL. Second, the study was conducted in a single developed Chinese province, limiting generalizability to other regions or countries with different pandemic responses and health systems. Third, the IDSHL scale primarily measures functional literacy and does not assess critical or interactive dimensions, nor digital health literacy directly. Fourth, self-reported data may be subject to social desirability bias, particularly during the pandemic. Fifth, while the Cronbach α values for the behavior (0.65) and skills (0.63) subscales are within the acceptable range for population surveillance given the scale’s multidimensionality [32], they are modest; observed changes in these domains should therefore be interpreted with some caution. Finally, the weighting procedure reduces but does not eliminate potential bias from sample composition changes.

### Implications

The findings carry several practical implications. First, public health authorities should leverage the postpandemic elevated baseline to broaden comprehensive infectious disease education, ensuring that respiratory-focused gains do not come at the expense of other threats (eg, hepatitis B and rabies). Second, targeted interventions are needed to reach older adults, less-educated populations, and ethnic minorities using hybrid (digital + traditional) channels. For example, in Zhejiang, community health workers could deliver face-to-face sessions on nonrespiratory diseases in rural senior centers, complemented by WeChat mini-programs with large-font, audio-assisted content, and monthly live question-and-answer sessions via local health official accounts. Third, routine surveillance of IDSHL should be sustained to detect declines in nonpandemic disease knowledge. By learning from the patterns observed during this global health crisis, short-term crisis responses can be translated into long-term, equitable public health preparedness [33].

### Conclusions

This 6-year study offers the first population-level evidence spanning the full COVID-19 pandemic cycle that a major public health crisis can be temporally associated with substantial and sustained gains in IDSHL. However, the observed improvements were not self-sustaining: they accelerated during the acute crisis phase and decelerated thereafter, and they did not automatically generalize to nonrespiratory diseases. Moreover, preexisting disparities by age, education, and ethnicity persisted. These findings underscore a core tension: crises can rapidly elevate targeted literacy, but maintaining and broadening those gains requires deliberate, ongoing investment. Policymakers should therefore view crisis-driven literacy improvements as a foundation for, not a replacement of, routine health education and equity-focused communication strategies.

## Supplementary material

10.2196/89958 Multimedia Appendix 1 Questionnaire on infectious disease–specific health literacy.

## Abbreviations

AAPC: average annual percent change
APC: annual percent changes
IDSHL: Infectious disease-specific health literacy
LSD: least significant difference
MCAR: Missing Completely At Random
NPI: nonpharmaceutical intervention
OR: odds ratio
